# Use of vital wheat gluten in aquaculture feeds

**DOI:** 10.1186/2046-9063-9-21

**Published:** 2013-11-16

**Authors:** Emmanuelle Apper-Bossard, Aurélien Feneuil, Anne Wagner, Frédérique Respondek

**Affiliations:** 1 Tereos Syral, Z.I. Portuaire, 67 390, Marckolsheim, France

**Keywords:** Vital wheat gluten, Fish, Protein, Digestibility, Performance, Health

## Abstract

**Summary:**

In aquaculture, when alternative protein sources of Fish Meal (FM) in diets are investigated, Plant Proteins (PP) can be used. Among them, Vital Wheat Gluten (VWG) is a proteinaceous material obtained from wheat after starch extraction. “It is mainly composed of two types of proteins, gliadins and glutenins, which confer specific visco-elasticity that’s to say ability to form a network providing suitable binding. This will lead to specific technological properties that are notably relevant to extruded feeds”. Besides these properties, VWG is a high-protein ingredient with an interesting amino-acid profile. Whereas it is rather low in lysine, it contains more sulfur amino acids than other PP sources and it is high in glutamine, which is known to improve gut health and modulate immunity. VWG is a protein source with one of the highest nitrogen digestibility due to a lack of protease inhibitor activity and to the lenient process used to make the product. By this way, addition of VWG in diet does not adversely affect growth performance in many fish species, even at a high level, and may secure high PP level diets that can induce health damages.

## Introduction

Intensive production of farmed fish, fed with compound feeds, has been largely increased mainly due to the growth of aquaculture production, but also because it is the most efficient way of production [1]. In such feeds, Fish Meal (FM) used to be the major source of proteins, especially for marine fish and salmonids [2]. Nevertheless, because of the limited amount of available FM in the market, its lack of sustainability, and its increasing price, its inclusion in diets has been progressively reduced. In order to achieve a low FM incorporation (below 10%) without impairing growth performance, an active research was conducted on plant proteins (PP), which represent an interesting alternative to FM. In this context, many studies were undertaken to evaluate the effects of replacing FM by different types of PP on fish growth and health [3-5]. Nowadays, several studies are exploring the possibility to decrease FM in a large extent by replacing them with a mixture of several PP [6].

Among the tested PP being considered to replace FM, Vital Wheat Gluten (VWG) is a PP source that has been given very promising results. Indeed, VWG can act as a pellet binder in extruded feed. Furthermore, it is a high quality protein source, highly digestible, with an interesting profile of amino-acids, especially a high level of glutamine. The action of antinutritional substances was not observed when

wheat gluten was used as fish meal replacement [7]. As a result, growth performance and feed efficiency are not modified when up to 50% of FM are replaced with VWG in the diet of salmon [8], trout [9], and sea bream [10]. Furthermore, when compared with soybean-meal (SBM), VWG does not damage gut structure in Atlantic salmon [8]. The use of VWG has to be emphasised in a global context tending to decrease incorporation of FM. Thus, this paper aims at reviewing the use of VWG, its functionalities and properties regarding farmed fish.

### Vital wheat gluten: technological and nutritional properties

VWG can be defined as “a cohesive, visco-elastic, proteinaceous material prepared as a co-product of the isolation of starch from wheat flour” [11]. It is obtained from wheat flour by washing the dough preparation under water, and then centrifugation. This process removes soluble fibres and starch fractions and recovers the insoluble protein fraction that is mainly constituted of two fractions defined as follows according to their solubility in aqueous alcohols: soluble gliadins and insoluble glutenins [12], which are balanced with equal amounts.

**Figure 1:**
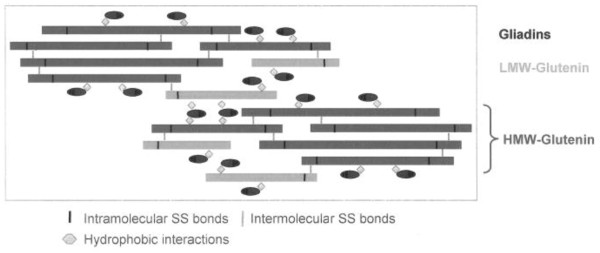
Representation of the gluten visco-elastic network and the place occupied by the gliadin and glutenin fractions within its structure.

Gliadins, which are monomeric proteins (intrachain disulfide bonds) with relatively low molecular weight (30 to 100 kDa), contribute to dough viscosity and extensibility, whereas glutenins, which are polymeric proteins (intra- and interchain disulfide bonds) with high molecular weight (100 kDa to more than 10,000 kDa), are both cohesive and elastic, being responsible for tenacity (resistance to deformation) and elasticity (Figure 1). Gliadins can be qualified as plasticiser for glutenins.

### A pellet binder

Due to its visco-elasticity, VWG can act as a pellet binder in extruded fish feed to partially replace starch or indigestible binders [8]. Indeed, the ability of fish to hydrolyse starch in the intestine and to regulate blood glucose concentration when the digestible carbohydrate level is high varies among species and is generally rather low [13], related to the enzymatic digestive capacity [14]. For example, the Atlantic salmon capacity to hydrolyse starch is low. Furthermore, administering glucose results in persistent hyperglycaemia in rainbow trout, common carp, red sea bream, yellowtail, and catfish [13], suggesting these species are not able to regulate glycaemia when fed with high level of digestible carbohydrates. That is why the starch amount is generally kept low in diets for fish, limiting its use as a technological binder.

Upon hydration, mixing, shearing, and heating (effects induced by the feed preparation, which is mostly done by extrusion-cooking), gliadins and glutenins interact in the dough through forces of various natures linked to their compositions: non-covalent bonds (hydrogen, ionic, and hydrophobic bonds) and covalent disulfide bonds [12] (Figure 1). Thus, gluten forms a strong cohesive network to entrap the other ingredients, providing adapted physical characteristics to the pellet in term of binding: improvement of the pellet hardness and pellet durability index (properties defined in Sorensen 2012). On our pilot extrusion line (Application Centre Tereos Syral, Marckolsheim, France), the production of two different salmon formulations containing 10 or 20% VWG (replacement of Soy Protein Concentrate (SPC) with VWG to switch from one formulation to the other) emphasised the importance of this trend: hardness and pellet durability index increase from 35 N to 48 N and from 97% to 98%, respectively, with increasing VWG. Similarly, a higher breaking force was induced by incorporating VWG compared to fish meal and SPC [15]. Moreover, VWG water insolubility reduces pellet breakdown [11], which can be interesting in cases where water stability must be high: for shrimp feeds (long residence time in water before eating) and in farms transporting the pellets from the weighing cell to the fish cages with water.

### A protein and amino acid source

The protein/energy ratio recommendation is higher for fish diets than for terrestrial vertebrates like pigs or poultry. Indeed, their basal energy requirements are lower than those of terrestrial vertebrates, due to the aquatic mode of life, poikilothermy, and ammonotelism [16]. As a result, the relative proportion of dietary protein in fish feed is higher than in terrestrial vertebrate feed. Dietary protein is then the major component of formulated fish feed. It is necessary to get both ingredients high in Crude Protein (CP) and high-quality proteins. Besides its technological properties, VWG has interesting nutritional values for fish feeds and is high in CP. The CP content is 80%, which is higher than in FM, SPC, Soybean Meal (SBM) or Pea Protein Concentrate (PeaPC) in which it represents between 44 and 72% [17].

**Figure 2:**
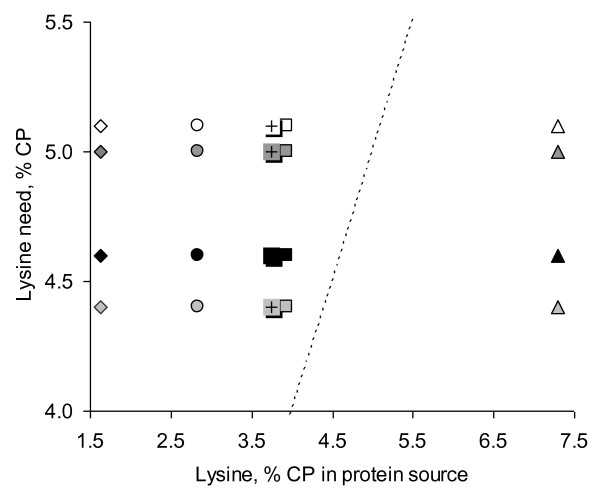
**Lysine requirements of several species of fish compared to content in different protein sources expressed as % of Crude Protein (CP).** White symbols: Nile tilapia requirement; dark grey symbols: Atlantic salmon requirement; black symbols: shrimp requirement; light grey symbols: European sea bass requirement. ◊: Vital Wheat Gluten; ○: Soybean Meal; +: Pea Protein Concentrate; □: Soy Protein Concentrate; ∆: Fish Meal. Dotted line: requirement of fish = content in protein source.

Regarding Essential Amino Acids (EAA), VWG is rather low in lysine, tryptophan, and arginine (Figure 2). Thus, a dietary supplementation with lysine in fish feed high in VWG is necessary. As example, while body of salmon contains 9.3 g lysine/100 g CP, VWG contains only 1.5 to 1.7 g/100 g CP [18]. Several experiments showed VWG can successfully replace a large part of FM when diets are supplemented with free lysine in salmonids [19,20]. A dose-effect relationship study involving VWG and lysine in rainbow trout showed better growth performance was obtained when FM was replaced with up to 50% VWG and when 0.29% to 0.58% lysine was added [20].

**Figure 3:**
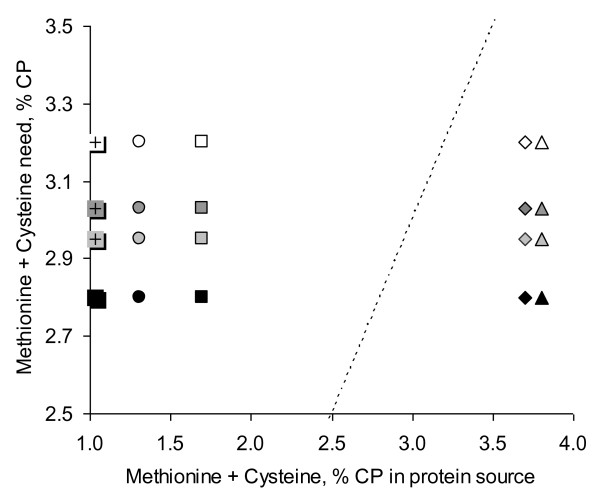
**Sulfur amino-acid requirements of several species of fish compared to content in different protein sources, expressed as % of Crude Protein (CP).** White symbols: Nile tilapia requirement; dark grey symbols: Atlantic salmon requirement; black symbols: Asian sea bass requirement; light grey symbols: European sea bass requirement. ◊: Vital Wheat Gluten; ○: Soybean Meal; +: Pea Protein Concentrate; □: Soy Protein Concentrate; ∆: Fish Meal. Dotted line: requirement of fish = content in protein source.

Beside the low amount of lysine, VWG contains a relatively high concentration of sulfur-containing amino acids, due to the numerous di-sulfur bounds (1.8% CP of methionine and 2.6% CP of cysteine), whereas PP sources are generally low in sulfur-containing amino acids (Figure 3). As examples, SBM and SPC respectively contain 1.4 and 1.3 g/100 g CP of methionine and 1.3 and 1.4 g/100 g CP of cysteine, which is lower than fish requirements. Furthermore, VWG is high in leucine, with about 7.9 g/100 g CP. Leucine is considered as the main amino acid triggering muscle protein synthesis and inhibiting proteolysis in mammals [21]. In particular, leucine can stimulate the PI3K-Akt-TOR pathway [22]. This mechanism may also occur in fish. Indeed, it is shown that in different species, amino acids regulate TOR signalling pathway [23]. Furthermore, supplementing media containing 0.6 mM leucine with an additional 2.5 mM leucine reduced rates of protein degradation of rainbow trout primary myocytes by 8% [24].

Regarding non-essential amino acids, VWG is high in glutamine: from 35 to 40% CP. Glutamine is a major substrate for all rapidly proliferating cells and plays an important role in maintaining intestinal trophicity [25] (Table 1). Also, glutamine is one of the most important energy substrate of enterocytes. Several studies recently demonstrated free glutamine significantly increases enterocyte and microvilli length of catfish gut [26], hybrid striped bass [27], and juvenile hybrid sturgeon [28] (Table 1). Besides these effects on gut morphology, glutamine also constitutes a major substrate for immune cells, thus modulating immune response [25] (Table 1). The serum non-specific immunity of juvenile hybrid sturgeon is modulated whereas lysozyme activity is increased in hybrid striped bass by adding glutamine in diets [27,29]. Moreover, glutamine also plays a role in eliminating free radicals as it acts as a precursor for the glutathione synthesis [30]. Such effects are reported for juvenile hybrid sturgeon [29] and hybrid striped bass [27]. Glutamine has proven to stimulate muscle synthesis in terrestrial vertebrates [31], but such results are not available for fish. However, dietary glutamine supplementation increases growth performance in juvenile hybrid sturgeon fed with a SPC-based diet [28], and in hybrid striped bass fed with a FM and SBM-based diet [27] (Table 1). Nevertheless, to our knowledge, the effects of VWG on immune response or on antioxidant status, compared to other PP sources, have not been investigated whereas its high amount of glutamine should be explored.

**Table 1:** Effects of adding glutamine on growth performance, gut structure and activity, and immune parameters in different species of fish

**Species**	**Physiologic status**	**Trial period (d)**	**Proteins in diets**	**Glutamine (Gln% in diet)**	**Main results**	**Reference**
Jian carp *Cyprinus carpio*	Juvenile iBW^1^ = 7.78 g	80	35% FM^2^; 20.5% Rice Gluten Meal; CP^3^ = 33% Substitution of glycine by Gln	0, 0.4, 0.8, 1.2, 1.6, 2.0	*Performance*	[32]
					Percent weight gain, feed efficiency, and productive protein linearly increase until 1.2% and reach a plateau afterwards.	
					*Gut morphology and activity*	
					Intestine fold height, and gut	
					length and weight linearly increase until 1.2% and reach a plateau afterwards.	
					Protease, lipase, AKP, and Na,K-ATPase activities linearly increase until 1.2% and reach a plateau afterwards in the 3 segments of gut.	
Hybrid sturgeon *Acipencer schrenckii* × *Huso dauricus*	Juvenile iBW = 22.38 g	56	20% SBM^2^; 10% Blood Meal; 20% FM; 8% Corn Protein; CP = 44.8% Substitution of SPC^2^ by HWG^2^ (0 to 5%) or by Aln-Gln (1%)	0, 0.63* 0, 0.3, 0.6, 0.9, 1.2, 1.5**	*Performance*	[28,29]
					Relative Growth Rate and feed efficiency linearly increase from 0 to 1.2% of Gln in HWG and increase with free Gln.	
					*Gut morphology and activity*	
					Intestine fold height, and gut length and weight linearly increase from 0 to 1.2% of Gln in HWG and increase with free Gln. Protease, amylase, lipase, and Na,K-ATPase activities linearly increase until 1.2% of Gln in HWG and increase with free Gln in the 3 segments of gut.	
					*Immune response and antioxidant status*	
					Glutathione peroxydase, glutataione, and superoxide dismutase concentrations linearly increase whereas malondialdehyde concentrations decrease until 1.2% Gln in HWG and with free Gln in intestine, hepatopancreas, and muscle.	
					Modulation of serum non-specific immune parameters according to a quadratic response with 0 to 1.5% Gln in HWG. Linearly increase of serum concentrations of C3 and C4 with addition of free Gln.	
Hybrid striped bass *Morone chrysops* × *Morone saxatilis*	Juvenile iBW = 4.12 g	56	34.6% Menhaden FM; 29.6% SBM; CP = 45% Substitution of glycine by Gln	0, 1, 2	*Performance*	[27]
					Specific Growth Rate and feed efficiency increase with 1% of Gln, not with 2%.	
					*Gut morphology*	
					No change in pyloric caeca. Enterocyte and microvillus heights in distal intestine and fold length in the proximal intestine increase with Gln. *Immune response* Higher serum lysozyme activity with 1% Gln. Increased extracellular superoxide anion production with 1% and 2% Gln.	
Channel catfish *Ictalurus punctatus*	Juvenile iBW = 6.1 g	70	13.4% casein; 3.76% gelatine; 7.1% of amino-acid premix; CP = 28%	0, 0.5, 1, 1.5, 2, 3	*Performance*	[26]
					No significant effect on growth performance. Increase of lipids in carcasses with 2 and 3% Gln.	
					*Gut morphology* Mucosal fold length, enterocyte height and microvilli height increase until 2% Gln in the 3 segments of intestine.	

^1^iBW: initial Body Weight; ^2^FM: Fish Meal, SBM: Soybean Meal, SPC: Soy Protein Concentrate, HWG: Hydrolysed Wheat Gluten ^3^CP: Crude Protein.

*Inclusion of 1% Aln-Gln; **: inclusion of 0 to 5% Hydrolysed wheat gluten.

### Wheat gluten in diets of fish

#### Highly digestible

**Figure 4:**
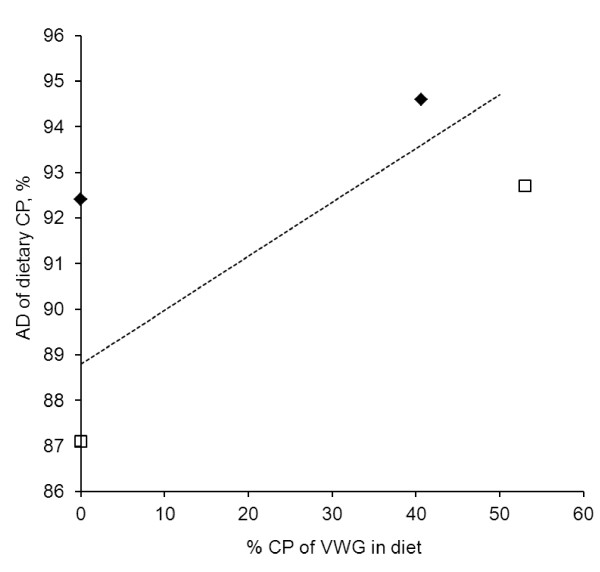
**Evolution of Apparent Digestibility (AD) of dietary Crude Protein (CP) according to the inclusion rate of Vital Wheat Gluten in diet, expressed as% of CP in Rainbow trout (adapted from **[20]**), European sea bass (adapted from **[32]**), and Atlantic salmon (adapted from **[8]**).** ♦: European sea bass; ------: Atlantic salmon (linear regression: AD_CP_ = 88.8 + 0.118× G, G being protein from VWG expressed as% of CP); □: Rainbow trout.

VWG is a highly digestible protein source for several species (Table 2). Apparent digestibility of 99% for CP when feeding a diet with 92.7% CP from VWG and 1.45% lysine is obtained in rainbow trout [33], whereas the apparent CP digestibility of VWG was estimated to approximate 100% [34]. In shrimp (*Litopenaeus vannamei*), the apparent CP digestibility is estimated at 98% [35], whereas it is 100% in Nile tilapia [17]. The apparent CP digestibility of VWG is higher than the apparent CP digestibility of numerous protein sources derived from plants and animals (Table 2). An inclusion of 15% VWG in a FM-based diet results in increasing CP digestibility in juvenile Nile tilapia. The same result is observed when around 30% VWG are added in a herring FM-based diet in Atlantic cod [36] or Australian silver perch [37] (Table 2). In salmonids, the apparent CP digestibility of diets rises with increasing inclusion rate of VWG [8,20,38], (Figure 4). In particular, in Atlantic salmon, the Apparent Digestibility (AD) of CP linearly increases from 88.6% with a FM-based diet to 93.6% when including 50% CP as VWG, and by extrapolating these results it was 100% with 100% CP from VWG [8].

**Table 2:** Apparent digestibility coefficient (ADC) of crude protein (CP) of diets with different protein sources depending on the inclusion rate and the species

**Species**	**Physiological status**	**Protein source**^**1**^	**Protein source in diet (%)**	**ADC of CP in diet or of ingredient (%)**	**Reference**
Coho salmon	Growing	VWG	30^2^	98.4	[34]
		SBM	100	96.4	
		Herring FM		96.6	
		Deboned meal		96.7	
		CGM		95.2	
		VWG		99.6	
		SBM		93.0	
		Herring FM		94.7	
		Deboned meal		95.1	
		CGM		91.9	
Rainbow trout	Growing	VWG	30^2^	98.6	[34]
		SBM	100	95.5	
		Herring FM		96.6	
		Deboned meal		97.2	
		CGM		97.4	
		VWG		100	
		SBM		90.1	
		Herring FM		94.6	
		Deboned meal		96.7	
		CGM		97.3	
Australian silver perch	Juvenile	VWG	29.7	99.8	[37]
		SBM		94.8	
		Australian FM		92.3	
		SDBM		90.2	
		CGM		95.4	
Gilthead sea bream	Growing	VWG	100^3^	96	[39]
		SPC		92	
		FM		86	
		CGM		90	
Nile tilapia	Juvenile	VWG	15	92.6	[40]
		SBM		90.5	
		DWD		88.9	
		FM		89.7	
Atlantic cod	Juvenile	VWG	29.8	99.9	[36]
		SBM		92.3	
		SPC		98.6	
		PeaPC		89.8	
		CGM		86.3	
		Herring FM		93.3	

^1^VWG: Vital Wheat Gluten; SBM: Soybean Meal; CGM: Corn Gluten Meal; SDBM: Spray-Dried Blood Plasma; SPC: Soy Protein Concentrate; PeaPC: Pea Protein Concentrate; FM: Fish Meal.

^2^Digestibility coefficients are given for both the diet with 30% protein source and for each protein source.

^3^Digestibility coefficients are indicated for each protein source.

**Figure 5:**
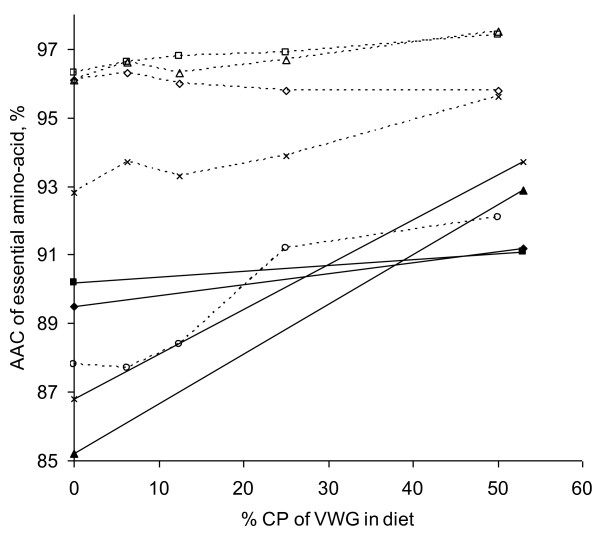
**Evolution of Apparent Availability Coefficient (AAC) of Arginine, Leucine, Lysine, Methionine, and Tryptophan according to the inclusion rate of Vital Wheat Gluten in diet, expressed as % of Crude Protein in Atlantic salmon (adapted from **[8]**) and Rainbow trout (diet supplemented with 0.29% Lysine; adapted from **[20]**).** Dotted line: Atlantic salmon; solid line: Rainbow trout. □: Arginine; ∆: Leucine; ◊: Lysine; ×: Methionine; ○: Tryptophan.

In these species, the apparent availability of EAA is also very high. In shrimp (*Litopenaeus vannamei*), the apparent availability of EAA from VWG is similar to or higher than the one of FM, except for lysine and cysteine. VWG has been reported to have equivalent or higher value with respect to apparent digestibility of protein and to apparent absorption of amino acids when compared to FM [8,34]. Specifically, the apparent digestibility of CP and apparent absorption of amino acids significantly increased with higher proportion of CP from VWG, except alanine and lysine [8] (Figure 5). The trend for a lower apparent absorption coefficient of lysine may be explained by a deviation between true and apparent lysine digestibility with increasing VWG, due to a relative higher excretion of endogenous lysine with high level of VWG.

The digestibility of energy from VWG is also higher than the digestibility of energy from FM in Atlantic salmon as well as in gilthead sea bream and Atlantic cod [8,39]. In contrast with these results, a decrease of energy and fat digestibility occurs when FM is replaced with SBM in Atlantic salmon [41] or by SPC and Corn Gluten Meal (CGM) in sea bream [39]. These differences may be attributed to a relative higher content of carbohydrates in soy protein ingredients or CGM compared to VWG. Carbohydrates are less available as an energy source to carnivorous fish for which the natural diet -and thus digestive metabolism- is based on lipids and proteins. VWG possesses both high gross energy and protein content and availability; it is then possible that a part of protein from VWG is used as an energy source in these species.

Phosphorus (P) is an essential component of fish diets. Not only it affects hard tissues but also intermediary metabolism and in turn, feed conversion ratio. In the same time, as the aquaculture industry grows, there are concerns regarding P in effluents. Thus, it is important to formulate diets containing adequate P for fish growth and ensuring minimal level of P in effluent. P bioavailability in PP sources is widely variable because of the presence of ANF. P availability is higher in VWG than in FM, SBM, SPC, and CGM for rainbow trout and coho salmon [34]. The content of ANF, such as phytic acid, is a major factor restricting the use of SBM and SPC in diets of fish [8]. The major vegetable part of P (60 to 70%) is bound to phytic acid, leading to a decrease in P availability in fish [42]. This phytic acid decreases the availability of cations such as zinc, magnesium, and calcium [43,44] or protein [42]. This problem does not appear for VWG, in which P content is relatively low but is highly available because of the absence of phytic acid.

### Low amount of fibres and no Antinutritional factors

The replacement of FM with other PP sources may result in increasing the level of fibres in diets [45]. Shrimp and fish do not have a high capacity to digest dietary fibres, and a high dietary level of fibres reduces digestibility and utilisation of other nutrients, acting as antinutritional factors (ANF) in carnivorous, as well as in herbivorous species. For instance, the level of dietary fibres must be less than 10% in rainbow trout otherwise leading to decreased growth and dry matter digestibility [46]. In tilapia, reducing weight gain is observed when fed diets with 10% soluble as well as insoluble fibres compared to a control diet [47]. The negative effect of dietary fibres on digestibility of dry matter or CP and growth is also demonstrated in common carp [48], Atlantic salmon [49], or European sea bass [50]. Such results have not been described when adding VWG in fish feed. Compared to other PP sources, VWG does not contain a high level of fibres. The amount of fibres in VWG averages 0.5 to 1% and is comparable to the amount found in FM. It is more than 10-fold lower than the level of fibres in SPC or SBM that averages 4.5% and 7.0%, respectively [17].

Additionally to increasing fibres, replacing FM by PP sources results in higher ANF content in diets. Such factors are defined as endogenous compounds of ingredients that may reduce feed intake, growth, nutrients digestibility and utilisation [51]. ANF are involved in the aetiology of diseases like enteritis, low lipid or protein digestibility and diarrhoea. These substances are numerous but the more relevant ones are phytic acids, enzyme inhibitors, lectin, saponins, phytoestrogens, phytosterols, and oligosaccharides. Due to their detrimental effects on zootechnical performance and nutrient utilisation, ANF must be taken into account when using high inclusion rates of PP for replacing FM [51].

The negative effects of ANF present in PP are different according to the type of PP, the process of production, and the dietary inclusion rate. Indeed, it appears that SBM induce histological and functional changes in gastro-intestinal tract (enteritis, increased susceptibility to bacterial infections, changes in absorptive cells …) in salmonids [52,53]. Inconsistent results are described for PeaPC: they can induce enteritis in Atlantic salmon [54]whereas no change on digestibility and mineral availability of rainbow trout was observed when replacing 50% FM by PeaPC [54]. Several experiments testing SPC reported no change on growth when comparing to a control, even if some modifications of gut structure were noticed [54]. Compared to other PP sources, no action similar to ANF has been described when VWG was used as FM replacement in different species [7].

Finally, due to the absence of fibres and ANF combined with a high amount of glutamine, adding VWG in diets may represent an opportunity in the current context in which PP are more and more present in diets. Indeed, diets with less than 5-10% FM are now formulated and used in practical diets for carnivorous fish. Such diets contain a mixture of several PP which have various nutritive properties. This strategy may lead to use complementary properties of different PP for balancing diets and diluting ANF.

### Growth performance

Because of its high digestibility and its absence of actions typical of ANF, replacement of a large proportion of FM with VWG results in similar growth performance and fish composition whatever the species are (Table 3). In Rainbow trout, VWG successfully substitutes more than 50% FM providing diets are supplemented with lysine without affecting protein and lipid composition of the pooled carcasses [9,20]. Furthermore, another experiment shows the inclusion of 14.5% VWG in diets does not adversely affect the flavour of fillets [55]. In Atlantic salmon, the replacement of 35% FM with VWG without supplementing by lysine results in similar final body weight and growth [8]. These authors estimate the replacement of FM with VWG without amino acid supplementation can go up to 50% based on the amount and the availability of lysine in VWG and on the requirement of fish. In European sea bass, substituting more than 50% FM with VWG in feeds does not impair palatability, growth performance, and nitrogen-energy retention [10]. In gilthead sea bream, the use of 88% CP from VWG not only replaced successfully FM but also produced better growth and feed conversion ratio, probably related to higher protein and energy intake of fish [39]. Such results are not obtained with CGM and SPC diets: feed efficiency and weight gain decrease when compared to FM (Table 4). In Nile tilapia fed with diets differing by their protein sources, the highest growth is reported for VWG, FM, and Soybean extract diet. In the same experiment, fish body composition is not significantly affected by diet ingredients [40] (Table 4). In shrimp, only few results are available but it seems the replacement of up to 20% marine protein with VWG does not modify feed efficiency [56].

**Table 3:** Effects of replacement of fish meal (FM) with vital wheat gluten (VWG) on growth performance, N and P losses, and whole body composition of several species of fish

**Species**	**Duration (d.)**	**FM (%CP**^**1**^**)**	**VWG (%CP)**	**Other PP**^**2**^**(%CP)**	**CP in diet (%)**	**Lys. Supp. (% diet)**	**iBW (g)**	**Main results**	**Reference**
Atlantic salmon	126	100	0	No	41.3	No	952	Similar growth - No modification in the mucosa of the posterior intestine.	[8]
		65	35	No	39.6	No	956		
Nile tilapia	49	88	0	No	32.5	No	56.4	Similar growth with VWG as with FM. Higher non faecal N losses with VWG than with FM. Lower faecal P losses than with FM. Same protein, fat and phosphorus body composition with VWG as with FM.	[40]
		58.3	32.8	No	34.7	No	55.6		
Rainbow trout	60	50	23.7	No	30.3	1.40	50	Similar growth with FM diet and when FM is totally replaced with a mixture of VWG and crystalline amino acids.	[9]
		0	77.0	No	31.6	2.50	50		
Rainbow trout	65	72	0	SBM^3^: 23;	45.5	No	23.7	Better growth and feed efficiency with VWG and 0.29% Lys than with the FM diet. Greater apparent net protein utilisation and protein efficiency ratio. No modification of protein and lipid content in carcasses.	[20]
		31	53	WM^3^: 5SBM: 6; WM: 5	46.6	0.29	23.7		
European sea bass	96	100	0	No	49.9	No	23.9	Weight gain slightly lower in fish fed with 70% VWG and no change in feed conversion ratio. Same faecal, retention and metabolic losses of N.	[10]
		50	50	No	49.7	0.20	23.9		
		30	70	No	49.9	0.80	23.9		
Sea bream	85	95	0	No	44.8	No	41.6	Significantly higher weight gain and feed efficiency with VWG than with FM. Same productive protein value and higher energy retention value with VWG than with FM. Same protein, lipid, energy, and phosphorus body composition with VWG as with FM.	[39]
		0	88	No	45.1	2.10	41.2		

^1^CP: Crude Protein; ^2^PP: Plant Protein; ^3^SBM: Soybean Meal; WM: Wheat Middlings.

**Table 4:** Effects of Vital Wheat Gluten (VWG) or other Plant Protein (PP) sources on growth performance of different fish species

**Species**	**Duration, d.**	**Tested Protein**^**1**^	**Protein, %CP**^**2**^	**%CP in diet**	**Lys. Supp. (% diet)**	**iBW**^**3**^**(g)**	**Main results**	**Reference**
Nile tilapia	49	FM	88	32.5	No	56.4	Highest feed efficiency and growth for FM, VWG, and SBE. No significant modifications in body composition for dry matter, protein, fat, P, and energy. Highest ash body content for DWD, SCP, and FM.	[40]
		VWG	32.8	34.7	No	55.6		
		SBM	23.9	30.5	No	55.6		
		SBE	34.2	35.7	No	54.3		
		DW	18.5	27.5	No	60.2		
		SCP	20.9	29.1	No	54.8		
Sea bream	85	FM	95	44.8	No	41.6	Better weight gain with VWG, before FM and before SPC and CGM. Same productive protein value and higher energy retention value with VWG as with FM.	[39]
		VWG	88	45.1	2.10	41.2		
		SPC	98	43.9	No	41.7		
		CGM	88	45.8	2.10	39.2		

^1^CP: Crude Protein; ^2^FM: Fish Meal; VWG: Vital Wheat Gluten; SBM: Soybean Meal; SBE: Soybean Extract; DW: Duckweed; SCP: single-cell protein; SPC: Soy Protein Concentrate; CGM: Corn Gluten Meal; ^3^iBW: initial Body Weight.

### Health

Even if the proportion of PP increases in fish feeds, and despite removing most of the indigestible carbohydrates, it still remains challenging when using plant protein concentrates at very high levels to replace up to 80% FM. Indeed, there are profound effects on metabolism, immunity, and gut health. In Rainbow trout and gilthead sea bream, protease activity is reduced with higher content of PP source until 100% FM are replaced [57]. A total replacement of FM with a mixture of PP exerts a positive antioxidant effect by enhancing the glutathione metabolism in gilthead sea bream [58]. It also modulates immune system, notably by decreasing complement levels in blood [58]. The effects of high dietary concentrations of soybean proteins on the non-specific immune response are also reported in rainbow trout with inconsistent results [59,60]. Gut and liver morphology is also modified when replacing FM with up to 50% PP in gilthead sea bream and rainbow trout [58,61]. All these results are complex and not always consistent. Furthermore, quantifying the relative effect of each PP in order to balance a diet high in PP remains difficult since current knowledge is still limited. Further investigations are needed to establish the effects of each PP and their interrelationships.

In this field, there is evidence in terrestrial animals showing that VWG is an interesting source of proteins for maintaining gut health and stimulating the immune system. In piglets, weaning is a highly stressful period characterised by an immediate and transient drop in feed intake, by modifications of various aspects of small intestine morphology and function (villi atrophy, drop in enzyme activity, local inflammation); and may lead to intestinal disorders and diarrhoea [62]. As for fish feed, replacement of animal proteins is investigated in starter feed. Replacing 6% FM or 6% Whey Protein Concentrate, considered as good protein sources for piglets, with 6% SBM or Rice Protein Concentrate results in diminishing villi height [63]. On the contrary, replacing 10% FM with 9.5% VWG does not negatively affect villi height, which is even numerically higher with VWG than with FM, with a similar feed intake [64]. These authors conclude that the positive effect of VWG on villi height may be attributed to the high amount of glutamine rather than feed intake. In fish, to our knowledge, very few data are available. Nevertheless, the replacement of 15% FM by SBM leads to pathological changes in the distal intestine of Atlantic salmon while the replacement of 35% of FM by VWG does not result in such damages, , probably related to the absence of ANF and to the high content of glutamine in VWG [8]. Furthermore, replacing 5% SPC with 5% Hydrolysed Wheat Gluten (HWG) results in improved performance, gut structure and activity, and immune response in juvenile hybrid sturgeons [28,29]. The results obtained with 3 to 5% HWG are similar to the results obtained when adding 1% glutamine, so that the authors conclude the effect of HWG on health may be due to the high amount of glutamine. The determination of the glutamine effect in native *vs* hydrolysed Wheat Gluten has not been investigated in fish and leads to inconsistent results in terrestrial animals, with either difference [64] or not [65]. These data suggest VWG can be a PP source limiting the negative impact on fish health when fed with high PP levels.

### Lipid metabolism

Lipid metabolism may be affected when fish are fed with high PP levels. It is important to take into account lipid metabolism in farmed fish since these factors may affect flesh quality. Adding high levels of PP sources in diets modulates lipid metabolism pathways when comparing to FM diets in several species [66]. Plasma cholesterol decreases in European sea bass (replacement up to 95% FM [67], in gilthead sea bream (replacement up to 100% FM; [58]) and in whole fillet of large rainbow trout (replacement up to 100% FM; [68]) when fish are fed with high amount of a blend composed of several PP sources. Also, a modification of fatty acid desaturation or elongation, with a strong increase of PUFA n-6 and a slight decrease of PUFA n-3 in fillets of large rainbow trout as in muscle of European sea bass are described [68,69]. Recently, a different lipid distribution between dorsal muscle and liver was observed when European sea bass is fed with up to 70% CP with different PP sources without modifying overall adiposity and mesenteric fat [69].

Several mechanisms are proposed to explain such effects. First, there is evidence from different animal models indicating the effect of varying the dietary protein source on fat content could depend on changes in the activity of liver lipogenic enzymes. In rats, the differential effect of dietary proteins on plasma cholesterol level is mainly associated with sulfur amino acids including in the protein [70]. More generally, differences in the amino acid composition of diets including plant *vs* animal proteins have been mentioned as a major factor in the mechanisms modulating *de novo* fatty acid synthesis [71]. Secondly, an increased PP source in diets is related to increased starch level so that both effects are confounded. A significant correlation between starch, glycaemia, and the activities of such lipogenic effects are already underlined in sea bream [72]. Thirdly, some ANF like isoflavones contained in soy proteins are shown to affect lipid metabolism and lower plasma cholesterol [73].

The effects of PP sources on lipid metabolism seem to be various, regarding the properties of PP source itself, i.e. the amino acid composition, the content of starch and the ANF. A dietary plant-protein substitution affects hepatic metabolism of rainbow trout but that the metabolic effects of PP replacement in fish feed varied with PP source [74]. In the different experiments previously cited, a blend of several PP sources was tested so that it is not possible to conclude which PP is the most involved compared to the others on the modulation of lipid metabolism and to define potential interactions between PP. Interestingly, when compared extruded or pelleted wheat gluten-based diets in European sea bass, no clear postprandial patterns of plasma cholesterol were discernible and cholesterol with VWG diet was only slightly, but not significantly, lower than plasma cholesterol of fish fed with FM diet [38]. Effects of up to 70% VWG as replacement of FM, a blend of VWG and pea, and a blend of VWG and SBM have been compared [69]. The plasma cholesterol and triacylglycerol concentrations were not significantly modified by VWG or VWG and pea but decreased with the blend of VWG and SBM, suggesting soy proteins play a major role in decreasing cholesterol and triacylglycerols in blood. However, VWG diets as soy-protein diets were found to decrease PUFA (n-3) and to increase PUFA (n-6) in muscle. These results underline PP have probably different routes of action on lipid metabolism.

## Conclusion

The use of VWG in fish feed offers different opportunities in term of technological properties. Furthermore, because of its nutritional properties, the replacement of a large part of FM with VWG is well-established and does not adversely affect feed intake, growth, feed and nutrient efficiency, and overall composition of different species of fish. Nowadays, research is refining and is moving towards understanding the impact of PP-based diets on fish health and metabolism. The knowledge of the pattern of each PP has to be completed by the knowledge of the effects of several PP in a same diet, their possible interactions, in order to optimise the formulation and the choice of ingredients. Indeed, using the correct mixture of PP provides not only the possibility of limiting damages, but also offers an interesting possibility to enhance antioxidant status and to modulate the immune response. In this way, the use of VWG in a diet containing high amount of PP may contribute to secure diets and to preserve health of fish.

## Competing interests

The authors declare that they have no competing interests.

## Authors’ contributions

EAB, AF, AW and FR participated in the sequence alignment and drafted the manuscript. All authors read and approved the final manuscript.
